# Roles of low-density lipoprotein receptor-related protein 1 in tumors

**DOI:** 10.1186/s40880-015-0064-0

**Published:** 2016-01-06

**Authors:** Peipei Xing, Zhichao Liao, Zhiwu Ren, Jun Zhao, Fengju Song, Guowen Wang, Kexin Chen, Jilong Yang

**Affiliations:** Department of Bone and Soft Tissue Tumor, Tianjin Medical University Cancer Institute and Hospital, Tianjin, 30060 P. R. China; National Clinical Research Center for Cancer, Tianjin Medical University Cancer Institute and Hospital, Tianjin, 30060 P. R. China; Department of Epidemiology and Biostatistics, Tianjin Medical University Cancer Institute and Hospital, Tianjin, 30060 P. R. China

**Keywords:** Low-density lipoprotein receptor-related protein 1, Tumorigenesis, Invasion, migration, Proliferation, apoptosis, Signaling pathway, MicroRNA, Fusion gene

## Abstract

Low-density lipoprotein receptor-related protein 1 (LRP1, also known as CD91), a multifunctional endocytic and cell signaling receptor, is widely expressed on the surface of multiple cell types such as hepatocytes, fibroblasts, neurons, astrocytes, macrophages, smooth muscle cells, and malignant cells. Emerging in vitro and in vivo evidence demonstrates that LRP1 is critically involved in many processes that drive tumorigenesis and tumor progression. For example, LRP1 not only promotes tumor cell migration and invasion by regulating matrix metalloproteinase (MMP)-2 and MMP-9 expression and functions but also inhibits cell apoptosis by regulating the insulin receptor, the serine/threonine protein kinase signaling pathway, and the expression of Caspase-3. LRP1-mediated phosphorylation of the extracellular signal-regulated kinase pathway and c-jun N-terminal kinase are also involved in tumor cell proliferation and invasion. In addition, LRP1 has been shown to be down-regulated by microRNA-205 and methylation of *LRP1* CpG islands. Furthermore, a novel fusion gene, *LRP1*-*SNRNP25*, promotes osteosarcoma cell invasion and migration. Only by understanding the mechanisms of these effects can we develop novel diagnostic and therapeutic strategies for cancers mediated by LRP1.

## Background

Low-density lipoprotein receptor-related protein 1 (LRP1, also known as CD91), a large endocytic receptor initially described by Herz et al. [1] in 1988, belongs to the low-density lipoprotein receptor (LDLR) superfamily. LRP1 is the most multifunctional member of this superfamily [1, 2]. Many translational studies have shown that LRP1 is involved in two major physiological processes: endocytosis and regulation of signaling pathways [3–5]. LRP1 mediates the endocytosis of a diverse set of extracellular ligands that play important roles in tumor progression [6]. In addition, LRP1 can initiate and regulate diverse signaling pathways, including mitogen-activated protein kinase (MAPK), insulin receptor (IR), serine/threonine protein kinase (AKT), extracellular signal-regulated kinase (ERK), and c-jun N-terminal kinase (JNK) pathways [7, 8]. Notably, these two physiological processes play an important role in multiple biological activities, including regulation of cell growth and differentiation, modulation of cytoskeletal organization angiogenesis, cell migration, and regulation of protease degradation for tissue invasion. Hence, in this review, we focus on recent translational studies that describe the roles of LRP1 in cancer cell proliferation, apoptosis, migration, invasion, and angiogenesis.

## From structure to function

**Fig. 1:**
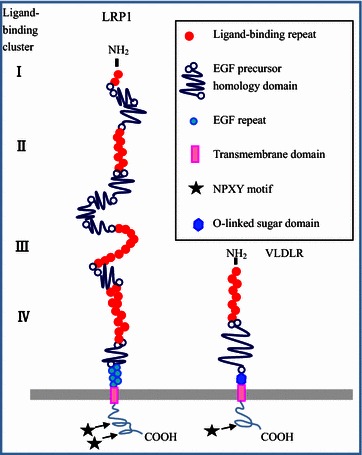
Structures of low-density lipoprotein receptor-related protein 1 (LRP1) and very low-density lipoprotein receptor (VLDLR). This Figure is modified from Figure 1 of a review written by Herz et al. [2]. The large extracellular ligand-binding subunit of LRP1 consists of 4 different ligand-binging clusters that contain 2, 8, 10, and 11 cysteine-rich ligand-binding repeats, respectively (*red dots*). Each cluster contains 1–4 epidermal growth factor (EGF) precursor homology domains, which include 2 cysteine-rich EGF repeats (blue circles) and 6 YWTD domains (*blue lines*). The cytoplasmic tail of LRP1 contains 2 NPXY motifs (*marked with an asterisk*). In addition, the cytoplasmic tail of LRP1 has 1 YXXL motif. Compared with LRP1, VLDLR is smaller. In addition, the O-linked sugar domain of VLDLR is enriched with serine and threonine residues (*blue hexagon*). Moreover, the cytoplasmic tail of VLDLR contains only 1 NPXY motif. Thus, the structure of LRP1 determines the endocytosis of ligands

The LDLR superfamily includes many members, such as LRP1, megalin (LRP2), LRP3, LRP4, LRP5, LRP6, very low-density lipoprotein receptor (VLDLR), and apolipoprotein E (apoE) receptor type 2 (apoER2, also known as LRP8) [9]. These proteins share five structural domains: (1) ligand-binding type cysteine-rich repeats, (2) epidermal growth factor (EGF) receptor-like cysteine-rich repeats, (3) YWTD domains, (4) a transmembrane domain, and (5) a cytoplasmic tail that harbors up to three NPXY motifs (Fig. 1) [2]. LRP1 consists of a large extracellular ligand-binding subunit (515 kDa), transmembrane subunit, and short cytoplasmic tail (85 kDa) [10]. Structurally, LRP1 is made up of 31 class A cysteine-rich repeats and an extracellular domain comprised of 4 ligand-binding clusters (I–IV), which have 2, 8, 10, and 11 complement-type repeats, respectively (Fig. 1) [11]. Domains II and IV mediate the binding of LRP1 to more than 40 ligands, including apolipoprotein, proteinases, proteinase-inhibitor complexes, bacterial toxins, viruses, the blood coagulation factor VIII, and various extracellular matrix proteins such as matrix metalloproteinases (MMPs) and urokinase-type plasminogen activator (uPA) [2, 12]. In contrast to LDLR, VLDLR, LRP2, and apoER2, the cytoplasmic tail of LRP1 contains 1 YXXL motif and 2 NPXY motifs (Fig. 1). The NPXY motifs function as the endocytosis signal for the LDLR family, and the YXXL motif serves as the dominant signal for rapid endocytosis of LRP1 [13, 14]. Therefore, the primary function of LRP1 is to bind ligands via the extracellular ligand-binding domain and promote their internalization via its cytoplasmic tail. Furthermore, LRP1 delivers bound ligands to the endosomal/lysosomal compartment for release and then returns to the cell membrane for reuse [15]. In addition to the endocytosis of multiple ligands, more recent studies have reported that LRP1 interacts and activates a diverse set of signaling proteins. Recently, Mantuano et al. [5] reported that some proteins can bind to LRP1 and activate LRP1-dependent signaling molecules such as ERK1/2. Within this context, proteins such as tissue-type plasminogen activator (tPA), apoE, and activated α_2_-macroglobulin (α_2_M) function as ligands of LRP1 [5]. In addition, substantial evidence indicates that LRP1 can activate various signaling pathways in response to particular ligands that bind to its extracellular domain by other mechanisms. For example, LRP1 binds to the platelet-derived growth factor (PDGF) receptor and forms a receptor-ligand complex that then activates the MAPK signaling pathway by driving endocytosis of the complex [4]. LRP1 has also been shown to activate signaling pathways by changing the levels of related receptors such as the uPA receptor (uPAR) [16]. Importantly, deregulation of the above signaling pathways has been implicated in tumor cell invasion and proliferation [4, 17]. Therefore, it is likely that, in addition to clearing the extracellular environment, LRP1-mediated endocytosis of ligands contributes to the delivery of cancer-promoting signals in cells. Consequently, LRP1 is suggested to be involved in two major cell processes: endocytosis and modulation of signaling pathways, which play diverse biological roles in cancer cells. Taken together, the unique structure of LRP1 contributes to its multi-functionality.

## Association between LRP1 and cancer susceptibility

The relationship between LRP1 and a variety of cancers has been reported. For example, Benes et al. [18] observed polymorphic alleles of C766T in exon 3 of *LRP1* gene and a marked increase in the frequency of the *LRP1* T allele in patients with breast cancer compared with control populations (0.21 versus 0.15, *P* = 0.01963), suggesting that the T allele of C766T in the *LRP1* gene may increase the risk of breast cancer. In addition, *LRP1* gene amplification has been found in astrocytoma, with 68 % of high-grade astrocytomas (grade IV) exhibiting high expression of LRP1, compared with the lack of LRP1 expression observed in 91 % of normal brain tissues [19]. Moreover, compared with low-grade astrocytoma, malignant glioma has been characterized with significantly higher levels of *LRP1* mRNA and protein [20]. In addition, increasing levels of LRP1 expression is commonly observed in the majority of endometrial carcinomas, suggesting that LRP1 is involved in the formation of endometrial carcinoma [21]. Tumorigenesis is a multistep process that involves cancer cell proliferation, angiogenesis, invasion, and migration [22]. Therefore, it is likely that LRP1 is involved in modulating some of these processes.

## The role of LRP1 in regulating cancer cell invasion and migration

### LRP1-mediated regulation of MMP expression promotes cancer cell migration and invasion

**Fig. 2:**
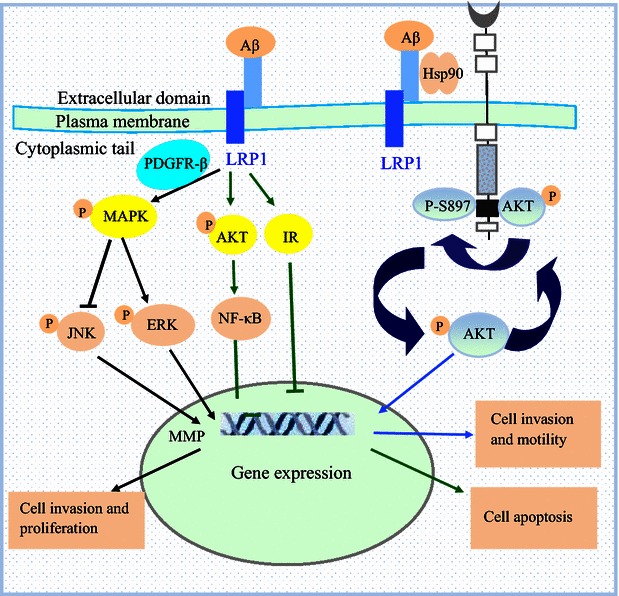
Low-density LRP1-mediated cell signaling pathways. LRP1 regulates several signaling pathways in a phosphorylation-dependent manner. These pathways are involved in several processes of tumorigenesis and progression. Binding of LRP1 to platelet-derived growth factor (PDGF) receptor activates the mitogen-activated protein kinase (MAPK) signaling pathway, which subsequently activates the extracellular signal-regulated kinase (ERK) pathway and inhibits c-jun N-terminal kinase (JNK), resulting in cancer cell invasion and proliferation. In addition, ERK increases the transcriptional levels of matrix metalloproteinase (MMP)-2 and MMP-9, which drive cancer cell invasion. LRP1 activates the serine/threonine protein kinase (AKT) signaling pathway and insulin receptor (IR), which inhibits cancer cell apoptosis. LRP1 binds to the novel extracellular heat shock protein 90 (eHsp90) and forms an eHsp90-LRP1 signaling complex, which stimulates glioblastoma multiforme cell motility and invasion via AKT-dependent phosphorylation at S897 (p-EphA2S897). The eHsp90 signaling regulates AKT activation and facilitates recruitment of LRP1 to the receptor tyrosine kinase EphA2

It is well known that MMPs, especially MMP-2 and MMP-9, are significantly involved in the invasion and metastasis of several types of human tumors [23, 24]. Recently, Song et al. [25] reported that LRP1 regulates the expression of MMP-2 and MMP-9, thereby promoting the migration and invasion of U87 human glioblastoma cells. In their study, knockdown of LRP1 using *LRP1* small interfering RNA in the human glioblastoma cell line U87 led to a decrease in cell migration and invasion, which suggests that the expression level of LRP1 is associated with migration and invasion capabilities of these cancer cells. Using LRP1-deficient and LRP1-overexpressing mouse embryonic fibroblasts cells, Song et al. [25] confirmed that LRP1-regulated MMP-2 and MMP-9 expression was involved in cell migration and invasion. In addition, they found that the level of phosphorylated ERK was decreased in LRP1-silenced cells, whereas other signaling pathways remained unchanged, suggesting that LRP1 regulates the expression of MMP-2 and MMP-9 via an ERK-dependent signaling pathway (Fig. 2) [25–27].

Other mechanisms have also been shown to contribute to LRP1-mediated MMP-dependent cancer cell invasion and migration. One study showed that the binding of the serine protease inhibitor protease nexin-1 (PN-1) to LRP1 stimulates MMP-9 expression, thereby promoting metastasis of breast cancer [28]. Similarly, α_2_M was shown to activate proMMP-2 via binding to LRP1 and promote the migration of human Müller glial cells [29]. However, α_2_M can also inhibit cell invasion through the clearance of pepsin from the extracellular environment [30, 31]. Thus, the role and mechanism of α_2_M in cell invasion and migration requires further investigation.

### LRP1-dependent activation of ERK and inhibition of JNK promote cancer cell invasion

Another mechanism by which LRP1 promotes cancer cell invasion is via activation of ERK and inhibition of JNK signaling pathways, which are two major MAPK pathways (Fig. 2). One study found that the level of phosphorylated ERK-1/2 was selectively decreased in LRP1-silenced cancer cells, whereas the level of phosphorylated JNK-1/2/3 was increased in cells, suggesting that LRP1 may serve as an intracellular signaling regulator by regulating the ERK and JNK signaling pathways [17]. Furthermore, these results are consistent with those of previous studies performed in fibroblasts, Schwann cells, and other non-tumor cells [32, 33]. In addition, the ERK signaling pathway has been shown to be activated during tumor cell invasion, and the invasion of JNK-overexpressing tumor cells is reduced, suggesting that ERK activation and JNK inhibition are required to promote tumor cell invasion [17]. Notably, the initial activation of the MAPK pathway is driven by the binding of LRP1 to PDGFR-bet in endosomes (Fig. 2) [4]. Future studies will be required to investigate the location of MAPK binding to LRP1.

### Role of the eHsp90-LRP1 signaling complex in tumor cell invasion and migration

Gopal et al. [20] identified a novel extracellular heat shock protein 90 (eHsp90) that can bind to LRP1 and form an eHsp90-LRP1 signaling complex in glioblastoma multiforme (GBM) (Fig. 2). The eHsp90-LRP1 complex was shown to regulate activation of the receptor tyrosine kinase EphA2, which is frequently overexpressed in most GBMs and stimulates GBM cell motility and invasion following activation by AKT-dependent phosphorylation at S897 (p-EphA2S897) (Fig. 2). In addition, positive feedback signaling from eHsp90 further activates AKT and promotes recruitment of LRP1 to EphA2 (Fig. 2), thereby strengthening this signaling pathway and highlighting a novel mechanism of LRP1-mediated regulation of tumor cell migration and invasion. In addition, the aforementioned mechanism is augmented during cellular hypoxia, which subsequently enhances the eHsp90-LRP1 signaling axis due to increased expression of LRP1 and eHsp90 [20, 34]. Collectively, these studies provide novel insight into how eHsp90-LRP1 promotes tumor cell migration and invasion and may lead to new therapeutic approaches to suppress the aggressiveness of GBM.

### Controversial data concerning the role of LRP1 in cancer cell invasion and migration

An increasing number of studies have confirmed that LRP1 promotes cancer cell invasion and migration. However, some investigations suggest that low expression of LRP1 can also promote tumor cell progression. uPA has been shown to be involved in tumor progression in a variety of cancers [35]. In human thyroid cancer cells, inhibiting LRP1 expression or increasing the level of uPA has been shown to enhance tumor cell invasion, specifically driving metastasis from lymph nodes and the lung [36]. Thus, low expression of LRP1 on the cell surface couples to increased expression and bioactivity of uPA and promotes tumor cell invasion [36]. Similarly, in fibroblasts, it has been reported that LRP1 inhibits cell migration by decreasing the cell surface abundance of uPA [16]. The mechanism of this function involves uPA binding to uPAR and activating plasminogen, which initiates a hydrolysis cascade of related proteins that drives degradation of the extracellular matrix. These processes facilitate tumor cell invasion through cell boundaries and hence, play a critical role in the migration of tumor and non-tumor cells [16]. Furthermore, a recent study has demonstrated that high expression of LRP1 is associated with a low metastatic potential of hepatocellular carcinoma (HCC) [37]. In addition, this study showed that inhibition of LRP1 increased the expression and bioactivity of MMP-9 in HCC cells. These results suggest a negative association between LRP1 and MMP-9 expression in HCC [37], which is in contrast to the observations made in U87 human glioblastoma cells [25]. Similar examples have also been found in other types of cancers, such as human endometrial carcinoma [38], breast and prostate cancers [39], lung cancer [40], and Wilm’s tumors [41]. Therefore, these data suggest that LRP1 may have a dual role in cancer cell invasion and migration, which potentially depends on the different cell type and specific extracellular microenvironment.

## The role of LRP1 in regulating cancer cell apoptosis and proliferation

Mounting evidence suggests that LRP1 plays an important role in cell proliferation and apoptosis. Fuentealba et al. [8] found that knockdown of LRP1 by short hairpin RNA in neurons significantly decreased the levels of Caspase-3 activation, AKT phosphorylation, IR signaling, and apoptosis. Caspase-3 is a key enzyme in cell apoptosis, suggesting that LRP1 may also inhibit cancer cell apoptosis by regulating the activation of Caspase-3. Moreover, the important role of LRP1 in activating the AKT and IR signaling pathways has been confirmed in the forebrain of LRP1-ablated mice, which were characterized with decreased cell apoptosis, suggesting that LRP1 inhibits apoptosis by activating the IR and AKT signaling pathways (Fig. 2) [8]. Subsequent studies have revealed the mechanism by which LRP1 activates the AKT signaling pathway and that α_2_M can bind to LRP1 and activate AKT in Schwann cells [32]. However, the specific LRP1-mediated mechanism of activation remains unclear. In addition, one study reported that LRP1 is involved in PDGF-mediated activation of ERK phosphorylation, which can increase smooth muscle cell and fibroblast proliferation [4]. Thus, LRP1 can regulate cell apoptosis, proliferation, invasion, and migration.

## Unique role of monocyte- and macrophage-associated LRP1 in tumorigenesis and tumor progression

Monocytes and macrophages promote cell invasion, metastasis, and angiogenesis in multiple cancers [42, 43]. In the initial step of tumor formation, macrophages create an inflammatory environment that promotes the growth of tumor cells. These growth-promoting effects on tumors are caused by the expression of multiple factors such as tumor necrosis factor-α and interleukin-6 [44, 45]. Moreover, macrophages produce EGF, which activates migration of tumor cells [46]. In addition, macrophages have been implicated in tumor angiogenesis. Accumulating studies reported that LRP1 in monocytes and macrophages plays a critical role in cancer progression by acting as a regulator of inflammation [45]. Staudt et al. [47] implanted PanO2 pancreatic carcinoma cells into mice that harbored LRP1-deficient myeloid cells and found that monocytes were strongly recruited to carcinoma cells. In addition, LRP1-deficient, bone marrow-derived macrophages expressed high levels of macrophage inflammatory protein-1α/CCL3 [47]. These findings suggest a negative association between LRP1 expression and the recruitment of monocytes and macrophages. Moreover, tumor-associated, LRP1-deficient macrophages were characterized by the release of vascular endothelial growth factor (VEGF) into the tumor microenvironment [47]. The level of VEGF is closely associated with microvessel density and the malignant degree of tumors and is increasing in tumor tissues compared with non-tumor tissues [47], suggesting that VEGF may promote tumorigenesis by regulating LRP1 to enhance angiogenesis. Together, the roles of monocyte- and macrophage-associated LRP1 in the initiation and progression of tumor suggest that targeted regulation of the inflammatory environment may prevent the occurrence of tumors.

## The multiple roles of *LRP1* CpG island methylation

Accumulating evidence suggests that DNA methylation of CpG islands plays a critical role in the initiation and progression of multiple cancer types [48, 49]. The promoter region of *LRP1* is enriched with CpG islands that govern sensitivity of the *LRP1* gene to DNA methylation. Moreover, all constructs containing CpG sequences can increase transcriptional activity. Sonoda et al. [50] studied the methylation status of *LRP1* CpG islands in esophageal squamous cell carcinomas (ESCCs) and found that, when CpG was methylated completely, the transcriptional activity completely disappeared and the expression of LRP1 was silenced. Moreover, the methylation of *LRP1* CpG islands was frequently observed in ESCC cells compared with control cells. Furthermore, when the expression of LRP1 was recovered, the growth of these ESCC cells was inhibited [50]. These data suggest that *LRP1* CpG island methylation may be involved in tumor progression by controlling the expression of LRP1. In addition, methylation of *LRP1* CpG islands may represent a novel diagnostic marker for ESCC and other cancers.

## microRNA-205 (*miR*-*205*) and LRP1

MicroRNAs are small noncoding RNAs that play a critical role in regulating post-transcriptional gene expression in various human cancers and are involved in driving tumorigenesis via regulation of metastasis, invasion, cell proliferation, and apoptosis [51]. Song et al. [52] examined U87 glioblastoma and SK-LU-1 lung adenocarcinoma cells, which express high levels of LRP1, and found that LRP1 expression and cell migration were both suppressed in cells transfected with *miR*-*205*. They also demonstrated that *miR*-*205* suppressed *LRP1* gene expression via inhibition of translation. Moreover, endocytosis of α_2_M, a ligand of LRP1, was significantly suppressed in these cells [52]. Together, these data suggest that *miR*-*205* down-regulates LRP1 expression and its endocytic function and leads to suppressed tumor cell migration and invasion [52]. Song et al. [52] also revealed the mechanism by which *miR*-*205* causes *LRP1* gene silencing. *miR*-*205* binds to its complementary sequences in the 3′ untranslated regions of *LRP1*, which plays a role in regulating LRP1 expression. Consistent with this finding, Kajihara et al. [53] recently reported that *miR*-*205* down-regulated LRP1 expression and suppressed ERK phosphorylation, thereby inhibiting cell proliferation in dermatofibrosarcoma protuberans. Consequently, these results highlight a potential tumor suppressive function of *miR*-*205* via regulating the expression of LRP1.

## Novel *LRP1*-*SNRNP25* fusion gene in osteosarcoma

Recently, a novel fusion gene, *LRP1*-*SNRNP25*, was identified in human osteosarcoma by transcriptome sequencing, reverse transcription-polymerase chain reaction, and Sanger sequencing [54]. *LRP1* and *SNRNP25* are located on 12q and 16p, respectively. Sanger sequencing revealed that *LRP1*-*SNRNP25* is formed by linking exon 8 of *LRP1* to exon 2 of *SNRNP25*. Fluorescence in situ hybridization not only confirmed that the fusion gene was resulted from interchromosomal rearrangement but also identified the amplification of the *LRP1*-*SNRNP2*5 fusion gene. In addition, transfection of *LRP1*-*SNRNP25* into SAOS-2 human osteosarcoma cells showed that the expression of *LRP1*-*SNRNP25* promotes SAOS-2 cell migration and invasion [54, 55]. Importantly, this novel finding may lead to a new therapeutic approach for osteosarcoma.

## Conclusions

The ability of LRP1 to promote endocytosis and deliver cell signaling suggests that LRP1 may play multiple roles in tumorigenesis and tumor progression. In addition, LRP1 has dual effects on tumor cell invasion and migration, reinforcing that the tumor cell-specific activity of LRP1 must be studied in the future. Moreover, LRP1 can be regulated via methylation of *LRP1* CpG islands as well as *miR*-*205*, which further complicates the role of LRP1 in cancer cells. LRP1 also serves as a regulator of monocytes and macrophages. Recently, the roles of the immune system in the initiation and progression of multiple cancers have received increasing attention. Thus, there are multiple opportunities to develop new LRP1-related diagnostic and therapeutic approaches for many types of cancers.
